# Validation of E1L3N antibody for PD-L1 detection and prediction of pembrolizumab response in non-small-cell lung cancer

**DOI:** 10.1038/s43856-022-00206-4

**Published:** 2022-11-01

**Authors:** Lianxi Song, Liang Zeng, Huan Yan, Qinqin Xu, Qing Xia, Jian Lei, Xiaoyan Chen, Xiaoping Hu, Zhan Wang, Hong Liu, Nong Yang, Yongchang Zhang

**Affiliations:** 1 Department of Medical Oncology, Lung Cancer and Gastrointestinal Unit, Hunan Cancer Hospital/The Affiliated Cancer Hospital of Xiangya School of Medicine, Central South University, Changsha, 410013 China; 2 Graduate Collaborative Training Base of Hunan Cancer Hospital, Hengyang Medical School, University of South China, Hengyang, 421001 China; 3 Department of Medical Oncology, Qinghai Provincial People’s Hospital, Xining, 810000 China; 4 State Key Laboratory for Oncogenes and Related Genes, Shanghai Cancer Institute, Renji Hospital, Department of Oncology, Shanghai Jiao Tong University School of Medicine, Shanghai, 210025 China; 5 Department of Pathology, Hunan Cancer Hospital/The Affiliated Cancer Hospital of Xiangya School of Medicine, Central South University, Changsha, 410013 China; 6 Department of Dermatology, Xiangya Hospital, Central South University, Changsha, 410008 China

**Keywords:** Tumour immunology, Immunotherapy

## Abstract

**Background:**

The programmed death-ligand 1 (PD-L1) 22C3 assay is one of the approved companion diagnostic assays for receiving anti-programmed cell death ligand 1 (PD-L1) therapy. Our study evaluated the performance of E1L3N and 22C3 antibodies in estimating PD-L1 expression in non-small cell lung cancer (NSCLC).

**Methods:**

Our retrospective study included 46 patients diagnosed with unresectable EGFR/ALK/ROS1-negative NSCLC who received first-line pembrolizumab therapy between 2018 and 2021. PD-L1 immunohistochemistry of baseline tissue biopsy samples was performed using PDL1-E1L3N and PDL1-22C3 antibodies. The concordance between the PD-L1 assays and the treatment outcomes was assessed.

**Results:**

Using a tumor proportion score (TPS) cutoff of ≥1%, 67.4% of patients are evaluated to be positive using PDL1-E1L3N and 73.9% using PDL1-22C3. Using a TPS of ≥50% as the cutoff, 26.1% of patients are positive using PDL1-E1L3N and 30.4% using PDL1-22C3. The PDL1-22C3 and PDL1-E1L3N assays are highly concordant and reveal a correlation coefficient of 0.925 (*p* < 0.0001). Patients with PDL1-E1L3N TPS > 50% have a significantly higher objective response rate than patients with PDL1-E1L3N TPS < 1% (*p* = 0.047), with a similar trend observed for PDL1-22C3 (*p* = 0.051). Consistent with PDL1-22C3, patients with higher PDL1-E1L3N expression (≥50%, 1–49%) have longer progression-free survival than those with PDL1-E1L3N TPS < 1%.

**Conclusion:**

Our study provides clinical evidence on the concordance of PD-L1 TPS scores between clones E1L3N and 22C3. Moreover, the treatment responses to pembrolizumab are also comparable between the PDL1-E1L3N and PDL1-22C3. These findings indicate that E1L3N is a reliable and cost-effective assay and may serve as an alternative to 22C3.

## Plain language summary

To determine which patients might be suitable to receive immunotherapy, a type of cancer treatment that triggers the immune system to target the patient’s tumor, a PD-L1 test is sometimes used. This test looks at the levels of PD-L1, which indicate whether immunotherapy might work in the patient. One of the tests commonly used can be expensive and a reliable and cost-effective alternative is needed. Here, we compare the results of PD-L1 testing with that test with an alternative that is more cost-effective. We show that the two tests are highly concordant, and also demonstrate that the results using the alternative test are able to predict response to immunotherapy in patients with advanced non-small cell lung cancer. Our findings provide evidence that the alternative, more cost-effective test might be useful and reliable in the clinic.

## Abstract (web summary)

Song, Zeng, Yan et al. validate the E1L3N monoclonal antibody for the detection of PD-L1 in NSCLC samples. The authors show that PD-L1 scores assessed with E1L3N are concordant with the 22C3 assay and are associated with response to first-line pembrolizumab monotherapy.

## Introduction

The efforts invested in clinical research on non-small cell lung cancer (NSCLC) have led to the continuous advancement of therapeutic strategies and the availability of effective treatment options for NSCLC. Of these treatment options, cancer immunotherapy has achieved satisfactory results in terms of improving survival outcomes of patients with advanced NSCLC^1–4^. The mechanism of tumor immune escape mediated by the programmed cell death receptor 1 (PD-1)/programmed death-ligand 1 (PD-L1) has been comprehensively elucidated and widely recognized^5^.

PD-L1 is the main ligand for PD-1 and is a type I transmembrane protein involved in cellular regulation and immune response^6–8^. In NSCLC, lung tumor tissues has higher PD-L1 expression level than normal lung tissues and benign lung lesions^9^. In lung cancers, PD-L1 expression was positively correlated with the clinical stage, lymph node metastasis, smoking history of patients, and EGFR expression level; while being negatively correlated with the prognosis and survival of patients^10^. These findings suggest that high PD-L1 expression in NSCLC tissues is an important molecular marker of disease progression and plays an important role in reflecting the level of T-cell-mediated anti-tumor immune response in the tumor microenvironment^10^. Thus, PD-L1 expression level can be used as an independent prognostic indicator and an attractive target for the treatment of NSCLC^11–13^.

The immunohistochemistry (IHC)-based detection of PD-L1 expression is critical in predicting the response to immunotherapy of patients with solid tumors, including NSCLC^14^. IHC assays using clones 22C3, SP142, SP263, and 28-8 are clinically validated for detecting PD-L1 expression in NSCLC^15,16^. The US Food and Drug Administration (FDA) approval of the Dako 22C3 pharmDx assay as a companion diagnostic test for receiving pembrolizumab therapy was based on various KEYNOTE studies (ie, KEYNOTE-001/010/024/042) that primarily used 22C3 assay for identifying PD-L1 tissue expression in NSCLC^17–20^. However, due to its limited availability and relatively higher cost, PD-L1 testing using 22C3 antibody becomes a challenge for patients with financial limitations. Therefore, there is an urgent need for finding accessible and lower-cost alternatives to enable the routine implementation of PD-L1 testing. E1L3N is a rabbit monoclonal antibody that binds specifically to the epitope of PD-L1 at the Phe19-Thr239 position^21,22^. E1L3N was shown to have a similar affinity and avidity as that of 22C3. A benchmarking study demonstrated the consistency between E1L3N and 22C3 using standardized cell line tissue microarray^23^. Due to the lack of clinical evidence on the utility of E1L3N, we designed this study to investigate and compare its performance with the 22C3 assay. In this retrospective study, we report the clinical performance of PD-L1 testing using E1L3N antibody by comparing it with the gold standard 22C3 antibody. We further assessed their performance in predicting pembrolizumab’s efficacy in the treatment of patients with advanced NSCLC in clinical settings. Our findings demonstrate that E1L3N-based and 22C3-based PD-L1 assays were highly concordant and reflect a similar trend in predicting treatment responses to pembrolizumab monotherapy. These findings indicate that the cost-effective E1L3N assay may serve as an alternative to 22C3.

## Methods

### Patient selection

**Fig. 1:**
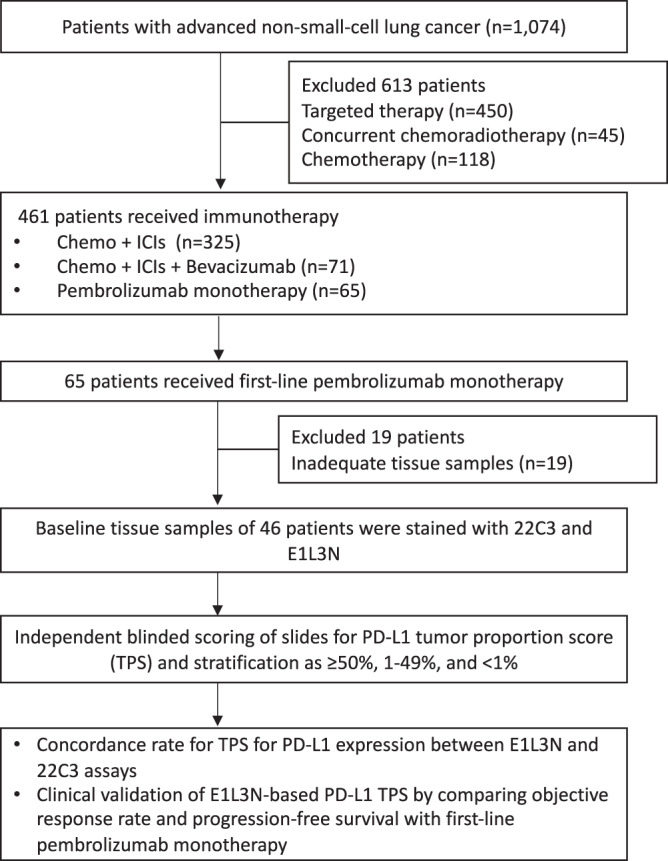
Flow chart of the study cohort. Abbreviation: Chemo + ICI chemotherapy plus immune checkpoint inhibitor therapy; TPS tumor proportion score.

Our retrospective study screened a total of 1074 patients who were diagnosed with NSCLC between January 2018 and November 2021 at Hunan Cancer Hospital, China. Eligible patients were ≥18 years of age with histologically or cytologically confirmed locally advanced or metastatic NSCLC without actionable mutations in *EGFR*, *ALK*, *ROS1*, *MET*, *RET*, *BRAF*, *KRAS*, *ERBB2*, or *NTRK*. Gene mutation status of the patients was assessed using next-generation sequencing (NGS)-based assay of either tumor or blood samples using any gene panel that interrogates at least the 8 classic NSCLC genes (i.e., *EGFR*, *ALK*, *ROS1*, *MET*, *RET*, *BRAF*, *KRAS*, and *ERBB2*)^24^. The disease stage was determined using the eighth edition American Joint Committee on Cancer (AJCC) staging system^25^. Patients were excluded if their tissue samples had inadequate tumor cells (<100 total number of tumor cells) on pathological evaluation. Patients who were being treated with pembrolizumab and neoadjuvant therapy or adjuvant therapy were excluded. All patients provided written informed consent for the use of their blood and tissue samples and clinical data for research purposes. The study protocol was approved by the institutional review board of Hunan Cancer Hospital (2021YYQ-SSB-131). This study was conducted in accordance with the Declaration of Helsinki. The flow diagram of the patient screening is summarized in Fig. 1.

### Study endpoints

The systemic response was assessed according to the Response Evaluation Criteria in Solid Tumors (RECIST, version 1.1). Tumor imaging was performed before initiating pembrolizumab treatment (baseline) and every 8 weeks until the date of treatment termination or confirmation of progressive disease (PD). Follow-up evaluations of brain metastases were carried out using Magnetic Resonance Imaging (MRI) or Computed Tomography (CT) scanning every two months for patients who presented with brain metastasis at initial diagnosis. The overall response rate (ORR) was defined as the proportion of patients achieving a partial response (PR) or a complete response (CR). Progression-free survival (PFS) was measured from the start of pembrolizumab treatment until PD. The primary endpoints were ORR and PFS. The cutoff date for survival analysis was November 10, 2021.

### Sample size estimation

This clinical study collected information on the efficacy of first-line pembrolizumab monotherapy. No control group was included in this analytical study. The acceptance standard was the median PFS (mPFS) with a 95% confidence interval (CI) of the test reagent (E1L3N) testing positive (tumor proportion score [TPS] ≥1%). The lower limit was greater than the lowest acceptable evaluation index (target value). In reference to the KEYNOTE-042 study, 637 patients with advanced NSCLC having TPS ≥1% assessed using 22C3-based PD-L1 IHC had an mPFS of 5.4 months (95%CI: 4.3, 6.2) with pembrolizumab monotherapy^20^. In our clinical study, the minimum target value for mPFS is 4.3 months and was based on the lower limit of the CI reported from the KEYNOTE-042 study. We estimated a sample size of at least 27 cases who were treated with pembrolizumab monotherapy with PD-L1 positivity of TPS ≥1% using the test reagent (E1L3N). Assuming that TPS ≥1% has a frequency of 60% among NSCLCs without actionable mutations, we estimated that 45 cases should be an optimal sample size for our study.

### PD-L1 IHC staining

Formalin-fixed paraffin-embedded (FFPE) tissue biopsy samples from 46 patients were prepared for IHC.

PD-L1 staining using E1L3N antibody was performed in Leica BOND-MAX fully automated IHC and ISH staining system (Leica Biosystems, Nussloch, Germany) following the manufacturer’s instructions and as follows: The FFPE tissue sections were deparaffinized using the Bond dewax solution, followed by heat-induced epitope retrieval at pH 9.0 using the Bond epitope retrieval solution 2 for up to 20 min at 100 °C. The slides were incubated with rabbit anti-PD-L1 E1L3N antibody (Cell Signaling Technology, Danvers, MA, USA) at 1:1000 and stained with the diaminobenzidine chromogen solution for 10 min at room temperature (Bond polymer refine detection kit, Leica Biosystems). The tissue sections were washed using Bond wash solution before counterstaining with hematoxylin (all Bond reagents, Leica Biosystems). Supplementary Fig. 1 shows the results from the exploratory assay for determining the optimal antibody dilution concentration and staining incubation time for the E1L3N antibody.

PD-L1 staining using 22C3 antibody was performed using Dako Autostainer Link 48 with a PT Link (Agilent Dako Omnis, Santa Clara, CA, USA) following the manufacturer’s instructions and as follows: The FFPE tissue sections were pretreated with EnVision FLEX target retrieval solution at a low pH of 6.1 after deparaffinization. Heat-mediated antigen retrieval was performed using PT Link at 97 °C for 20 min. Subsequently, the slides were incubated with 22C3 antibody (Agilent Dako Omnis, Santa Clara, CA, USA) at 1:100, stained for 30 min at room temperature, and visualized using PD-L1 IHC 22C3 pharmDx kit (Agilent Dako Omnis). The slides were counterstained with hematoxylin after washing with EnVision FLEX wash buffer (Agilent Dako Omnis).

### Assessment of PD-L1 expression

PD-L1 expression in tumor tissues was independently assessed and scored by two pathologists in a double-blinded manner. Cases that were found to be discordant for each assay were reassessed by both pathologists until an agreement was reached. A senior pathologist also reviewed the discordant cases for each assay to derive a definitive TPS. The final PD-L1 TPS for each assay was obtained by averaging the two TPS values from the two pathologists. PD-L1 expression levels for the two antibodies were then compared for each patient in an unblinded manner. PD-L1 protein expression in NSCLC was determined by using TPS, which was the percentage of viable tumor cells showing partial or complete membrane staining at any intensity. PD-L1 positivity was defined as TPS ≥ 1%. We further stratified PD-L1 positivity as moderate PD-L1 expression, which refers to TPS 1–49% and high PD-L1 expression, which was defined as TPS ≥ 50%. This scoring method was adapted from the KEYNOTE-042 trial^20^.

### Statistics and reproducibility

The study population was described in terms of frequencies for qualitative variables, or medians and associated ranges for quantitative variables. Chi-squared test was performed to calculate differences between subgroups for each variable and considered significant if the *p*-value was <0.05. The correlation between PD-L1 TPS assessed for E1L3N and 22C3 assays was calculated using Pearson Correlation Coefficient. Survival probabilities were estimated by the Kaplan-Meier method and survival curves were compared using the log-rank test. Statistical analyses were carried out using R software version 3.1.2.

### Reporting summary

Further information on research design is available in the Nature Research Reporting Summary linked to this article.

## Results

### Patient disposition and baseline characteristics

The clinicopathological characteristics of the patients stratified according to the different PD-L1 detection antibodies are listed in Table 1. Among the 46 patients who received first-line pembrolizumab monotherapy, 40 (86.9%) patients were men and 11 (27.5) were never smokers. The most frequent histologic diagnosis was squamous cell carcinoma, accounting for 58.7% (*n* = 27) of patients. Seven patients were detected with *KRAS* mutations (i.e., G12C, *n* = 4 and G12A, *n* = 3), 1 patient had *HER2* exon20 insertion mutation, 1 patient had *KIF5B-RET* fusion, 1 patient had *EGFR* exon 20 insertion mutation, and the other patients were not detected with actionable mutations. A majority (36/46, (78.2%) of patients in our cohort had stage IV disease, and the remaining 10 patients had IIIb/IIIc locally advanced stage disease. Five (10.9%) and 7 (15.2%) patients had either brain metastasis or liver metastasis at baseline examination, respectively.

**Table 1:** Clinical characteristics of the 46 patients with NSCLC who received first-line pembrolizumab treatment.

Characteristics	Total no. (%)
No. of patients	46
Age, years	
Median	65.3
Range	47–81
Sex	
Male	40 (86.9)
Female	6 (13.1)
Smoking history	
Never smoker	22 (47.8)
Former smoker	24 (52.2)
Histology	
Adenocarcinoma	18 (39.1)
Squamous carcinoma	28 (60.9)
ECOG performance status	
0–1	44 (95.6)
≥2	2 (4.4)
Stage	
IIIb/IIIc	10 (21.8)
IV	36 (78.2)
Brain metastasis	
Yes	5 (10.9)
No	41 (89.1)
Liver metastasis	
Yes	7 (15.2)
No	39 (84.8)
Treatment line	
First-line	46 (100)
Objective response	
PR	17 (36.9)
SD	16 (34.8)
PD	13 (28.3)
Objective response rate	36.9%

*ECOG* Eastern Cooperative Oncology Group, *PR* partial response, *SD* stable disease, *PD* progressive disease

### PD-L1 IHC assay

**Fig. 2:**
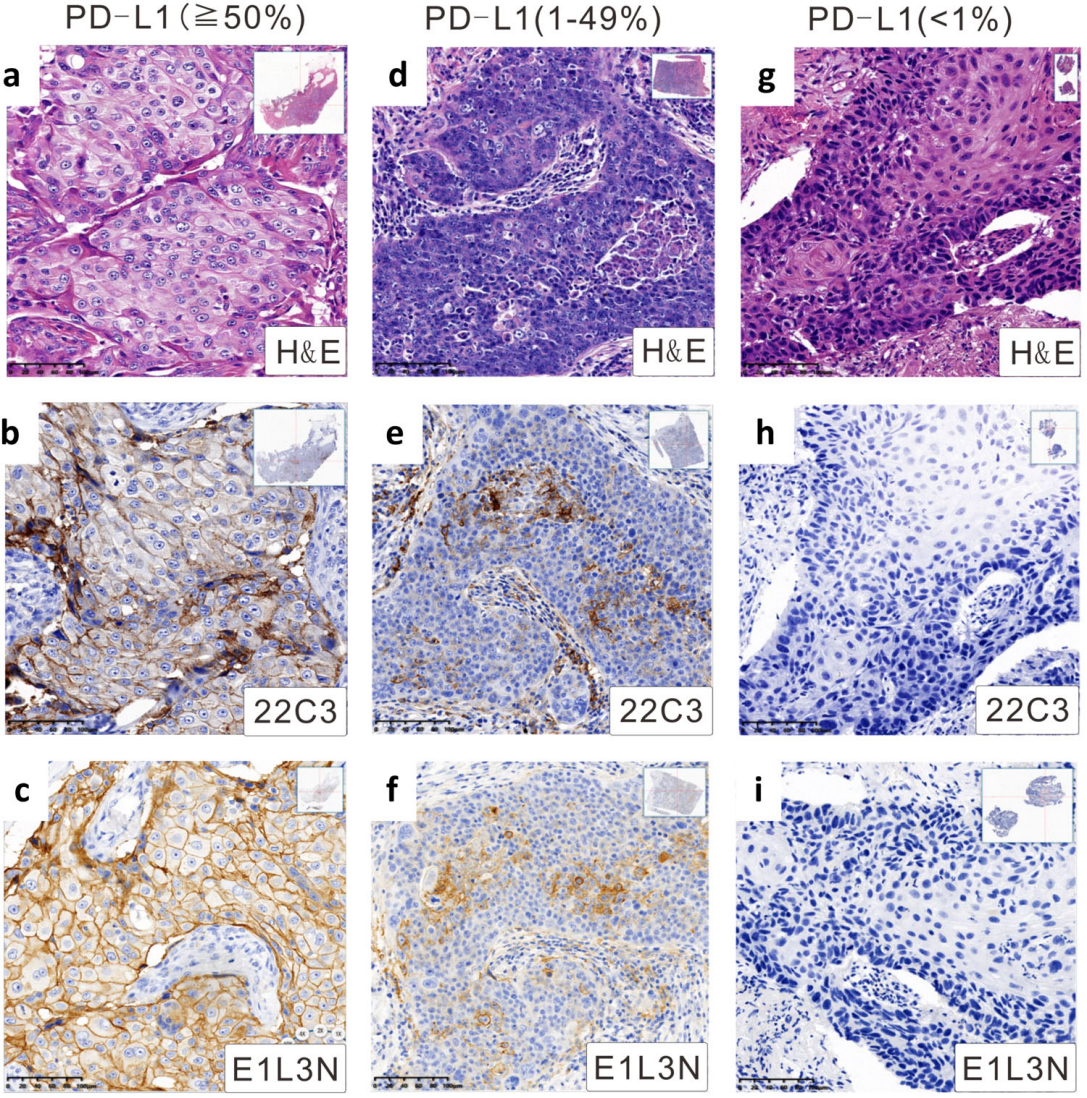
Representative micrographs for PD-L1 tumor proportion score (TPS). Representative micrographs for PD-L1 tumor proportion score (TPS) of ≥50% (**a**–**c**), 1–49% (**d**–**f**), and <1% (**g**–**i**). **a**, **d**, and **g** show the hematoxylin-eosin-stained slides. **b**, **e**, and **h** show the 22C3-stained slides. **c**, **f**, and **i** show the E1L3N-stained slides. Abbreviations: H&E hematoxylin-eosin; TPS tumor proportion score.

**Fig. 3:**
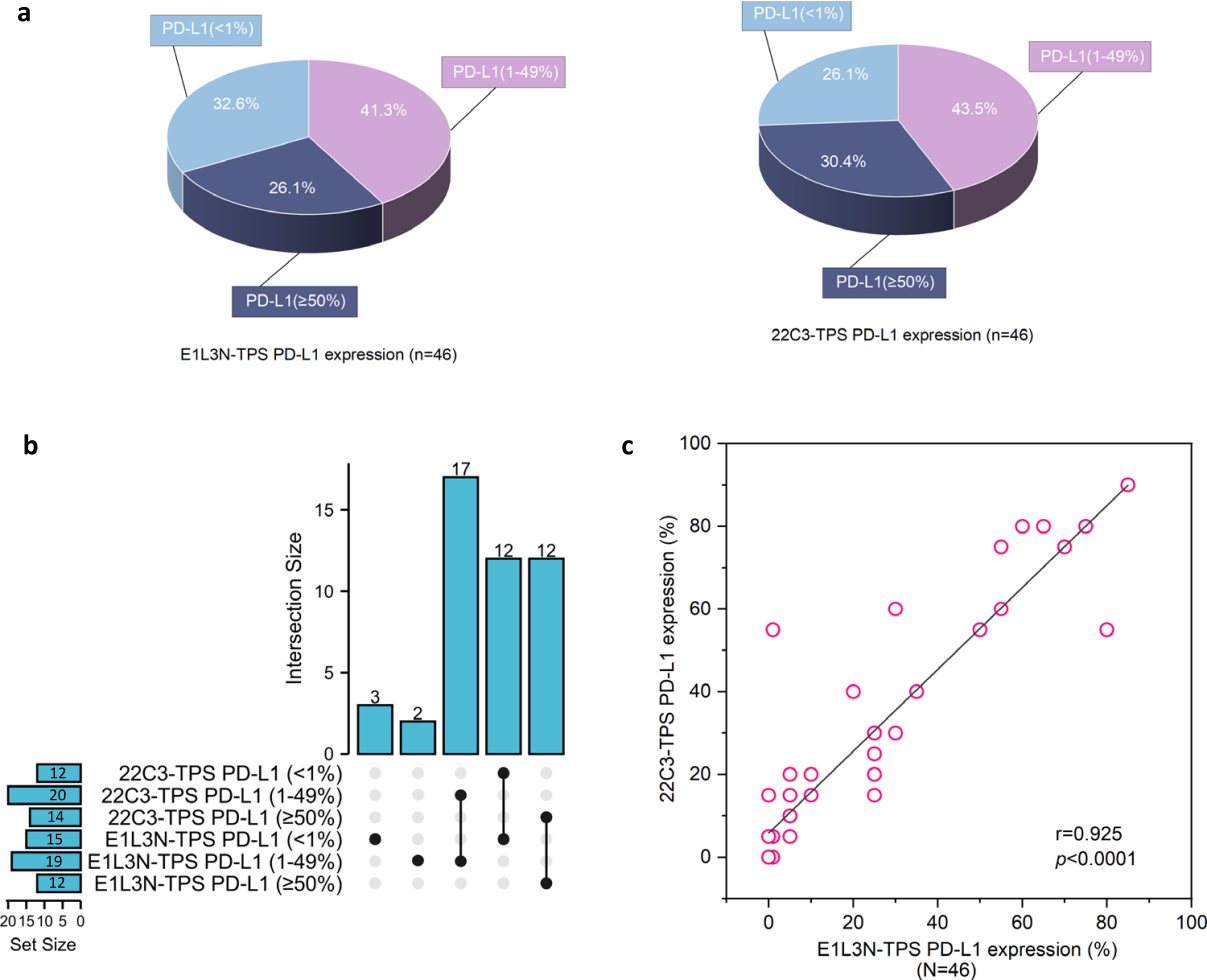
PD-L1 tumor proportion scores (TPS) were concordant between E1L3N and 22C3. **a** Pie chart showing the distribution of the cohort stratified according to PD-L1 TPS for E1L3N (left) and 22C3 (right). **b** UpSet plot illustrating the intersection/concordance between 22C3 and E1L3N TPS subgroups. The height of the columns corresponds to the numbers labeled on top of each column, which indicates the number of intersected data. The dots below the columns indicate the subgroup to which the data belongs to. The connected dots below the columns indicate the number of patients having concordant PD-L1 TPS categorized as <1% (*n* = 12), 1–49% (*n* = 11), and ≥50% (*n* = 7). The bar plot on the left side illustrates the sample size per subgroup. **c** Correlation plot showing the positive correlation between PD-L1 TPS scores for E1L3N assay (*x*-axis) and 22C3 assay (*y*-axis). The correlation between 22C3 TPS and E1L3N TPS was computed using Pearson Correlation Coefficient (*R*^2^). Abbreviations: TPS tumor proportion score.

Tumor cell labeling using 22C3 and E1L3N antibodies showed a range of intensities from partial to full circumferential membrane staining. The comparative analysis did not include the staining intensity, but the percentage of stained cells was included. Figure 2 depicts three NSCLC cases representing a range of PD-L1 staining intensities albeit concordant PD-L1 TPS assayed using the two antibodies. Figure 3a summarizes the distribution of our study cohort based on PD-L1 TPS at cutoffs of <1%, 1–49%, ≥50% for the two antibodies. Using a TPS ≥1% as the cutoff for PD-L1 positivity, E1L3N assay positivity was observed in 67.4% (31/46) of NSCLC cases, whereas 22C3 assay positivity was 73.9% (34/46). The PD-L1 expression detected by 22C3 and E1L3N antibodies was highly concordant (Supplementary Fig. 2), particularly at TPS < 1% (Fig. 3b). Relative to 22C3-based PD-L1 expression, 12 patients with TPS < 1%, 17 patients with TPS 1–49%, and 12 patients with TPS ≥50% had concordant PD-L1 status using E1L3N assay, resulting in a concordance rate of 89.1% (41/46) (Fig. 3b). Moreover, E1L3N-based PD-L1 TPS was positively correlated with 22C3-based PD-L1 TPS, with a Pearson correlation coefficient of 0.925 (*p* < 0.0001; Fig. 3c). The 5 patients with discordant PD-L1 TPS were as follows: 2 patients with 22C3-based TPS of ≥50% had E1L3N-based PD-L1 TPS of 1–49%, 3 patients with 22C3-based TPS of 1–49% had E1L3N-based PD-L1 TPS of <1%. Supplementary Table 1 lists the clinical details of the five patients with discordant PD-L1 TPS between the two assays. Supplementary Data 1 tabulates the PD-L1 TPS for both assays for each of the 46 patients.

### Quantitative PD-L1 TPS using E1L3N as a predictor of pembrolizumab response

Due to the heterogeneity of PD-L1 expression detected by different antibodies, we explored the relationship between PD-L1 TPS and patient outcomes to compare the clinical performance of these PD-L1 assays in identifying patients who may benefit from immunotherapy. All 46 patients received pembrolizumab monotherapy as first-line therapy. Based on RECIST criteria, 17 of 46 patients had tumor shrinkage of ≥30% with best response of PR (Supplementary Fig. 3), demonstrating an ORR of 36.9%. We stratified our cohort into three subgroups according to PD-L1 TPS as ≥50%, 1–49%, and <1%. It should be noted that pembrolizumab monotherapy was administered to 12 patients with PD-L1 < 1% due to the (i) absence of actionable mutations for targeted therapy eligibility and refusal to take chemotherapy or were poor candidates for chemotherapy (*n* = 10); and (ii) unknown PD-L1 expression status at the time of treatment (*n* = 2).

**Fig. 4:**
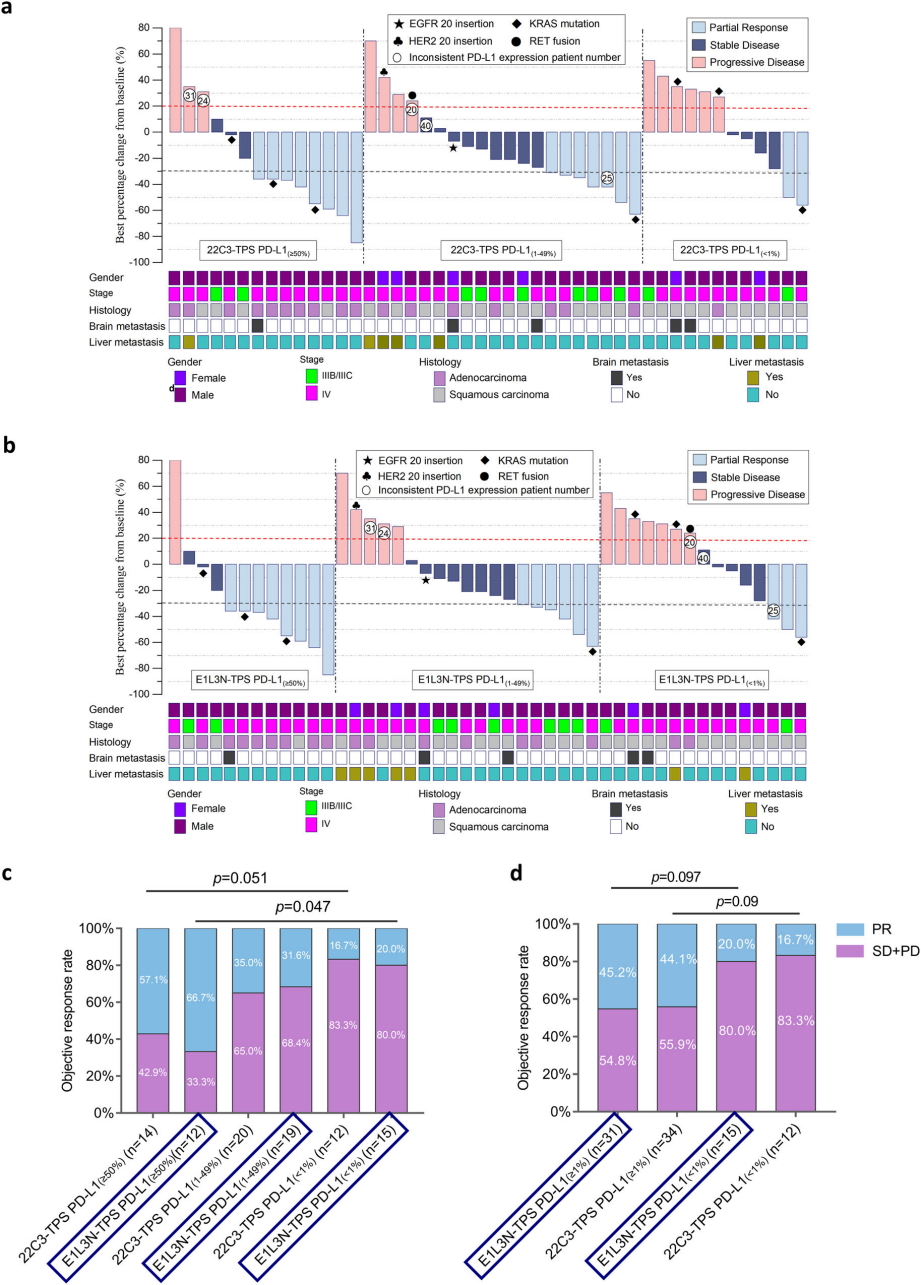
Treatment responses with first-line pembrolizumab monotherapy were consistent between E1L3N and 22C3. Waterfall plots showing the best change in tumor size relative to baseline for patients with tumor proportion scores (TPS) of ≥50%, 1–49%, and <1% assayed using 22C3 (**a**) and E1L3N (**b**). The encircled numbers indicate the patient number of patients with discordant 22C3 and E1L3N TPS subgroup. Best response and clinical details of each patient were annotated at the bottom of the waterfall plot and represented by different colors according to the legends. Red dotted line indicates the tumor size change of +20% as the cutoff for evaluating progressive disease. Green dotted line indicates the tumor size change of −30% as the cutoff for evaluating partial response. Component bar plots comparing the distribution of patients with partial response (PR) and stable disease (SD)/progressive disease (PD) stratified into either three (**c**) or two (**d**) subgroups according to TPS scores assayed using 22C3 and E1L3N. Abbreviations: PR partial response; SD + PD stable disease and progressive disease; TPS tumor proportion score.

**Fig. 5:**
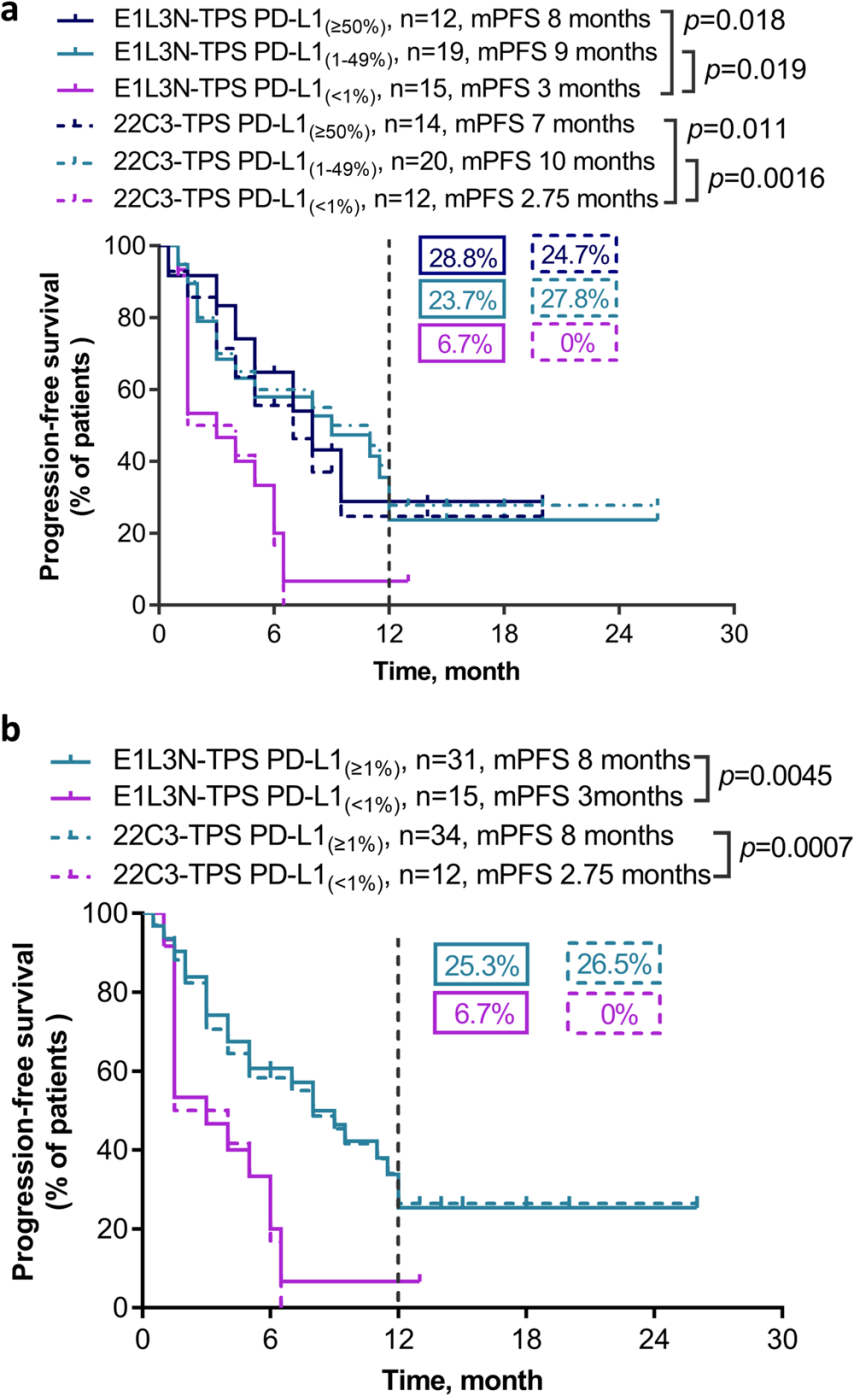
Progression-free survival with first-line pembrolizumab monotherapy was consistent between E1L3N and 22C3. **a** Kaplan–Meier survival curve comparing the progression-free survival of patients stratified into three subgroups according to TPS scores assayed using 22C3 and E1L3N as E1L3N-TPS PD-L1 ≥ 50% (*n* = 12), 1–49% (*n* = 19), and <1% (*n* = 15) and 22C3-TPS PD-L1 ≥ 50% (*n* = 14), 1–49% (*n* = 20), and <1% (*n* = 12). **b** Kaplan–Meier survival curve comparing the progression-free survival of patients stratified into two subgroups according to TPS scores assayed using 22C3 and E1L3N as E1L3N-TPS PD-L1 ≥ 1% (*n* = 31), and <1% (*n* = 15) and 22C3-TPS PD-L1 ≥ 1% (*n* = 34), and <1% (*n* = 12). The vertical black dotted line corresponds to the 1-year PFS. The boxed percentages correspond to the 1-year PFS of each subgroup. Abbreviations: mPFS median progression-free survival; TPS tumor proportion score.

Treatment responses had a similar pattern for 22C3 assay (Fig. 4a) and E1L3N assay (Fig. 4b) across PD-L1 TPS subgroups. We investigated the relationship between different PD-L1 expression levels and ORR. Patients with E1L3N-based PD-L1 TPS of ≥50% had significantly higher ORR than those with E1L3N-based PD-L1 TPS of <1% (66.7% vs. 20%; *p* = 0.047; Fig. 4c). Although no statistical difference was found for the clinical outcomes across PD-L1 TPS subgroups for 22C3 assay, the trend was consistent with the findings for E1L3N assay. A numerically higher ORR was observed among the patients with 22C3-based PD-L1 TPS of ≥50% than those with 22C3-based PD-L1 TPS of <1% (57.1% vs. 16.7%; *p* = 0.051; Fig. 4c, d). Furthermore, we also investigated the relationship between different PD-L1 expression levels and PFS. The swimmers plot shows PFS data for each of the 46 patients (Supplementary Fig. 4). Patients with E1L3N-based PD-L1 TPS of ≥50% (mPFS, 8 months vs. 3 months; *p* = 0.018; Fig. 5a) and 1–49% (mPFS, 9 months vs. 3 months; *p* = 0.019; Fig. 5a) had significantly longer PFS than patients with E1L3N-based PD-L1 TPS of <1%. Consistently, patients with 22C3-based PD-L1 TPS of ≥50% (mPFS, 7 months vs. 2.75 months; *p* = 0.011; Fig. 5b) and 1–49% (mPFS, 10 months vs. 2.75 months; *p* = 0.0016; Fig. 5b) than patients with 22C3-based PD-L1 TPS of <1%. The 1-year PFS rates with first-line pembrolizumab monotherapy were comparable between E1L3N and 22C3 for each of the three PD-L1 TPS subgroups (i.e., ≥50%; 1–49%; <1%). The 1-year PFS rates were 28.8, 23.7, and 6.7%, respectively, for E1L3N TPS subgroups and 24.7, 27.8, and 0%, respectively, for 22C3 TPS subgroups (Fig. 5b).

## Discussion

In 2019, the FDA has approved an extended indication for pembrolizumab as a first-line treatment in patients with metastatic NSCLC whose tumors had PD-L1 TPS ≥1%^1,18,26^. A series of KEYNOTE clinical trials demonstrated that IHC-based PD-L1 expression in tumor tissue was correlated with a better ORR and survival outcome with pembrolizumab^20,26–29^. The promising results from pivotal KEYNOTE-001/010/024/042 studies prompted the FDA and other regulatory agencies to approve pembrolizumab as the first- and second-line treatment in patients with NSCLC without EGFR or ALK alterations but had positive PD-L1 tissue expression^20,26–30^. KEYNOTE-001/010/024/042 studies selected the patients using the IHC-based PD-L1 22C3 assay with the Dako Autostainer platform^20,26–30^. This consequently resulted in the approval of the PD-L1 22C3 assay as the companion diagnostic assay for pembrolizumab therapy^19^. However, the limited availability and high cost of 22C3-based PD-L1 testing limited its routine use despite the acceptable performance of 22C3 antibody in predicting the therapeutic benefit of anti-PD1 therapy^18,31^. The higher cost of PD-L1 testing using 22C3 is due to the cost of the antibody and the use of a dedicated automated workflow from Dako. Efforts have been invested in exploring the use of other platforms or antibodies for PD-L1 testing, such as SP263 and 28-8^18,31,32^. Although the PD-L1 TPS were concordant between 22C3, SP263, and 28-8^32^, some studies have shown inter-clone variation in PD-L1 positivity using cutoffs of 1 and 50%, which were considered as clinically relevant cutoffs^33^. In this study, we evaluated the utility of E1L3N antibody to detect PD-L1 expression and the clinical outcomes associated with E1L3N-based PD-L1 positivity. Our findings showed that E1L3N-based PD-L1 TPS and corresponding treatment outcomes were highly correlated and consistent with 22C3-based PD-L1 TPS.

Our results indicated that E1L3N-based PD-L1 assay had a similar clinical performance as the gold standard 22C3-based PD-L1 assay based on their comparable ORR, PFS, and 1-year PFS rates. It was reported that a cutoff of ≥50% was predictive of response to first-line treatment with pembrolizumab^28^. Using a ≥50% cutoff to stratify our cohort, PD-L1 was categorized as positive in 26.1% of patients using the E1L3N assay and 30.4% of the patients using the 22C3 assay. PD-L1 expression levels between 22C3 and E1L3N showed technical equivalence based on their high concordance and positive correlation. However, we have also observed discordant PD-L1 TPS from 5 patients (10.9%). We speculate that the discordance in PD-L1 TPS between 22C3 and E1L3N assays was due to the inherent spatial heterogeneity of tumors that could result in the discrepancy in tumor content of the microsections used for these two assays. Another reason might also be related to the stronger affinity of the 22C3 antibody to the PD-L1 on tumor cells, which could be observed as the staining of more tumor cells resulting in higher PD-L1 expression level or TPS, as compared with other antibodies against PD-L1^17^.

Clinical trials with pembrolizumab, nivolumab, and atezolizumab have shown positive associations between clinical efficacy and level of tumoral PD-L1 expression as evaluated by PD-L1 IHC^17,34–36^. Although many studies with 22C3 and Ventana’s SP263 yielded overlapping results, they had shown discrepancies for the clinically relevant cutoffs (1 and 50%)^33,37,38^. Thus, we also evaluated the ORR of patients across different cutoffs for categorizing PD-L1 positivity. The clinical outcomes were generally consistent between E1L3N and 22C3 across TPS subgroups and showed that PD-L1 TPS of ≥50% using E1L3N was associated with a better ORR and PFS. Based on its high concordance with 22C3, E1L3N is a reliable and cost-effective alternative for the clinical detection of PD-L1 expression. As compared to the 22C3 assay, E1L3N antibody is cheaper and is not platform-dependent, which could allow most pathology laboratories to conveniently adopt this workflow for the routine screening of PD-L1 expression.

Several limitations were noted in this study. This study had no clinical outcome data except for the actual PD-L1 expression status; hence, the analytical sensitivity and specificity could not be calculated. Another limitation is the small sample size and potential patient selection bias due to the selection of patients who could afford the cost of 22C3 testing and pembrolizumab regimen. Randomized clinical studies with a larger sample size are needed to verify the result of this study. Our study also only investigated clinical responses to pembrolizumab monotherapy in the first-line setting. The use of E1L3N in predicting response to other PD-L1 or immune checkpoint inhibitors might need to be validated.

In conclusion, our study provided clinical evidence on the concordance and positive correlation of the E1L3N-based PD-L1 assay with the gold standard 22C3 assay. Moreover, the treatment responses to first-line pembrolizumab monotherapy were also comparable between the E1L3N and 22C3 assays. These findings indicate that E1L3N is a reliable and cost-effective assay that may serve as an alternative to 22C3. E1L3N-based assay could be developed for the routine screening of PD-L1 expression status.

## Supplementary information

Supplementary Information

Supplementary Data 1

Supplementary Data 2

Supplementary Data 3

Supplementary Data 4

Description of Additional Supplementary Files

Reporting Summary

## Data Availability

Source data is available as Supplementary Data 1 and available as Supplementary Data 2–4 spreadsheet files for Figs. 3–5. Other data (such as imaging data) are available from the corresponding author upon reasonable request.

## References

[1] Gadgeel S (2020). Updated analysis from KEYNOTE-189: Pembrolizumab or placebo plus pemetrexed and platinum for previously untreated metastatic nonsquamous non-small-cell lung cancer. J. Clin. Oncol..

[2] Paz-Ares L (2018). Pembrolizumab plus chemotherapy for squamous non-small-cell lung cancer. N. Engl. J. Med..

[3] Yang Y (2020). Efficacy and safety of sintilimab plus pemetrexed and platinum as first-line treatment for locally advanced or metastatic nonsquamous NSCLC: A randomized, double-blind, phase 3 study (Oncology pRogram by InnovENT anti-PD-1-11). J. Thorac. Oncol..

[4] Zhou C (2021). Sintilimab plus platinum and gemcitabine as first-line treatment for advanced or metastatic squamous NSCLC: Results from a randomized, double-blind, phase 3 trial (ORIENT-12). J. Thorac. Oncol..

[5] Lantuejoul S (2020). PD-L1 testing for lung cancer in 2019: Perspective from the IASLC pathology committee. J. Thorac. Oncol..

[6] Zou W, Wolchok JD, Chen L (2016). PD-L1 (B7-H1) and PD-1 pathway blockade for cancer therapy: Mechanisms, response biomarkers, and combinations. Sci. Transl. Med..

[7] Taube JM (2012). Colocalization of inflammatory response with B7-h1 expression in human melanocytic lesions supports an adaptive resistance mechanism of immune escape. Sci. Transl. Med..

[8] Talay O, Shen CH, Chen L, Chen J (2009). B7-H1 (PD-L1) on T cells is required for T-cell-mediated conditioning of dendritic cell maturation. Proc Natl Acad Sci USA.

[9] Larsen TV, Hussmann D, Nielsen AL (2019). PD-L1 and PD-L2 expression correlated genes in non-small-cell lung cancer. Cancer Commun..

[10] Chen X (2021). ILT4 inhibition prevents TAM- and dysfunctional T cell-mediated immunosuppression and enhances the efficacy of anti-PD-L1 therapy in NSCLC with EGFR activation. Theranostics.

[11] Shen X, Zhao B (2018). Efficacy of PD-1 or PD-L1 inhibitors and PD-L1 expression status in cancer: Meta-analysis. BMJ.

[12] Aguilar EJ (2019). Outcomes to first-line pembrolizumab in patients with non-small-cell lung cancer and very high PD-L1 expression. Ann. Oncol..

[13] Brody R (2017). PD-L1 expression in advanced NSCLC: Insights into risk stratification and treatment selection from a systematic literature review. Lung Cancer.

[14] Hwang DM (2021). Prevalence and heterogeneity of PD-L1 expression by 22C3 assay in routine population-based and reflexive clinical testing in lung cancer. J Thorac. Oncol..

[15] Hendry S (2018). Comparison of four PD-L1 immunohistochemical assays in lung cancer. J. Thorac. Oncol..

[16] Prince, E.A., Sanzari, J.K., Pandya, D., Huron, D. & Edwards, R. Analytical concordance of PD-L1 assays utilizing antibodies from FDA-approved diagnostics in advanced cancers: A systematic literature review. *JCO Precision Oncol.* **5**, 953–973 (2021). 10.1200/PO.20.00412 PMC8202559 34136742

[17] Hirsch FR (2017). PD-L1 immunohistochemistry assays for lung cancer: Results from phase 1 of the blueprint PD-L1 IHC assay comparison project. J. Thorac. Oncol..

[18] Marchetti A (2017). Multicenter comparison of 22C3 PharmDx (Agilent) and SP263 (Ventana) assays to test PD-L1 expression for NSCLC patients to be treated with immune checkpoint inhibitors. J. Thorac. Oncol..

[19] US-FDA. List of Cleared or Approved Companion Diagnostic Devices (In Vitro and Imaging Tools) https://www.fda.gov/medical-devices/in-vitro-diagnostics/list-cleared-or-approved-companion-diagnostic-devices-in-vitro-and-imaging-tools (2022).

[20] Mok TSK (2019). Pembrolizumab versus chemotherapy for previously untreated, PD-L1-expressing, locally advanced or metastatic non-small-cell lung cancer (KEYNOTE-042): A randomised, open-label, controlled, phase 3 trial. Lancet.

[21] Smith J (2016). Quantitative and qualitative characterization of Two PD-L1 clones: SP263 and E1L3N. Diagn. Pathol..

[22] Munari E (2019). PD-L1 expression in non-small cell lung cancer: Evaluation of the diagnostic accuracy of a laboratory-developed test using clone E1L3N in comparison with 22C3 and SP263 assays. Hum. Pathol..

[23] Martinez-Morilla S (2020). Quantitative assessment of PD-L1 as an analyte in immunohistochemistry diagnostic assays using a standardized cell line tissue microarray. Lab Invest..

[24] Zhang Y (2020). Detection of nonreciprocal/reciprocal ALK translocation as poor predictive marker in patients with first-line crizotinib-treated ALK-rearranged NSCLC. J. Thorac. Oncol..

[25] Lababede O, Meziane MA (2018). The eighth edition of TNM staging of lung cancer: Reference chart and diagrams. Oncologist.

[26] Garon EB (2015). Pembrolizumab for the treatment of non-small-cell lung cancer. N. Engl. J. Med..

[27] Reck M (2018). Pembrolizumab as first-line therapy for metastatic non-small-cell lung cancer. Immunotherapy.

[28] Herbst RS (2016). Pembrolizumab versus docetaxel for previously treated, PD-L1-positive, advanced non-small-cell lung cancer (KEYNOTE-010): A randomised controlled trial. Lancet.

[29] Reck M (2019). Updated analysis of KEYNOTE-024: Pembrolizumab versus platinum-based chemotherapy for advanced non-small-cell lung cancer with PD-L1 tumor proportion score of 50% or greater. J. Clin. Oncol..

[30] Kang SP (2017). Pembrolizumab KEYNOTE-001: An adaptive study leading to accelerated approval for two indications and a companion diagnostic. Ann. Oncol..

[31] Buttner R (2017). Programmed death-ligand 1 immunohistochemistry testing: A review of analytical assays and clinical implementation in non-small-cell lung cancer. J. Clin. Oncol..

[32] Ratcliffe MJ (2017). Agreement between programmed cell death ligand-1 diagnostic assays across multiple protein expression cutoffs in non-small cell lung cancer. Clin. Cancer Res..

[33] Munari E (2018). PD-L1 Assays 22C3 and SP263 are not interchangeable in non-small cell lung cancer when considering clinically relevant cutoffs: An interclone evaluation by differently trained pathologists. Am. J. Surg. Pathol..

[34] Liu L (2021). Efficacy and safety of first-line immunotherapy combinations for advanced NSCLC: A systematic review and network meta-analysis. J. Thorac. Oncol..

[35] Xu C (2018). Comparative safety of immune checkpoint inhibitors in cancer: Systematic review and network meta-analysis. BMJ.

[36] Kooshkaki, O. et al. Combination of ipilimumab and nivolumab in cancers: From clinical practice to ongoing clinical trials. *Int. J. Mol. Sci.* **21**, 4427 (2020). 10.3390/ijms21124427 PMC7352976 32580338

[37] Park Y (2020). PD-L1 testing in gastric cancer by the combined positive score of the 22C3 PharmDx and SP263 assay with clinically relevant cut-offs. Cancer Res. Treat..

[38] Sughayer MA, Alnaimy F, Alsughayer AM, Qamhia N (2019). Comparison of 22C3 PharmDx and SP263 assays to test PD-L1 expression in NSCLC. Appl. Immunohistochem. Mol. Morphol.: AIMM.

