# Supplementary material for: Validation of E1L3N antibody for PD-L1 detection and prediction of pembrolizumab response in non-small-cell lung cancer
Source: Commun Med (Lond). 2022 Nov 1;2:137. doi: 10.1038/s43856-022-00206-4 (PMC9626637; doi:10.1038/s43856-022-00206-4)
Supplement: Supplementary file 1 — Supplementary Information [file 43856_2022_206_MOESM1_ESM.pdf]

Supplementary Table 1. Five patients with discordant PD-L1 expression between 22C3 and E1L3N

Patient NO	Sex	Age	Histology	Stage	Brain Metastasis	Liver Metastasis	NGS gene alterations	PD-L1 TPS (22C3)	PD-L1 TPS (E1L3N)	Treatment line for pembrolizumab	Best response	PFS (months)	Best tumor size change from baseline (%)
31	M	61	ADC	IV	N	Y	Wild type	60%	30%	1L	PD	1.5	35
20	M	69	ADC	IV	N	N	*KIF5B-RET* fusion	5%	<1%	1L	PD	1.5	24
24	M	64	SC	IV	N	N	Not detected	55%	1%	1L	PD	3	31
40	M	81	SC	IV	N	N	Not detected	15%	0	1L	SD	3	11
25	M	59	SC	IV	N	N	Not detected	5%	0	1L	PR	13+	-42

1L, first-line; ADC, adenocarcinoma; M, male; N, no; NGS, next-generation sequencing; PD, progressive disease; PFS, progression-free survival; PR, partial response; SC, squamous cell carcinoma; SD, stable disease; TPS, tumor proportion score; Y, yes

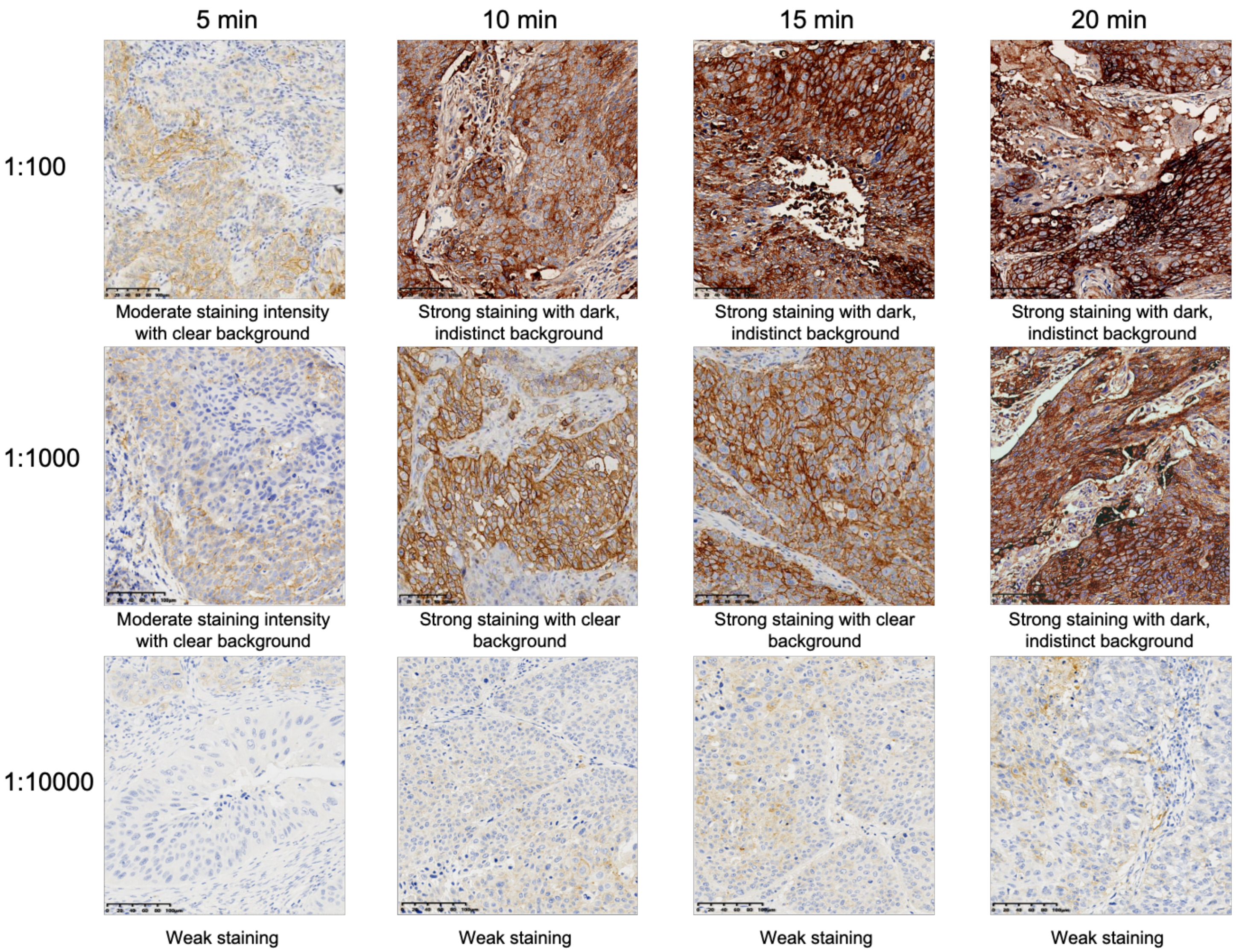

Supplementary Figure 1. Exploratory assays for E1L3N antibody staining conditions, including antibody dilution concentration and incubation time for the diaminobenzidine (DAB) chromogen solution. E1L3N antibody at varying dilution concentrations (i.e., 1:100, 1:1000, and 1:10,000) were stained at different incubation times (i.e., 5 minutes, 10 minutes, 15 minutes, and 20 minutes). Based on these results, the optimal staining condition was determined to be 1:1000 antibody dilution and 10-minute incubation with the DAB staining solution.

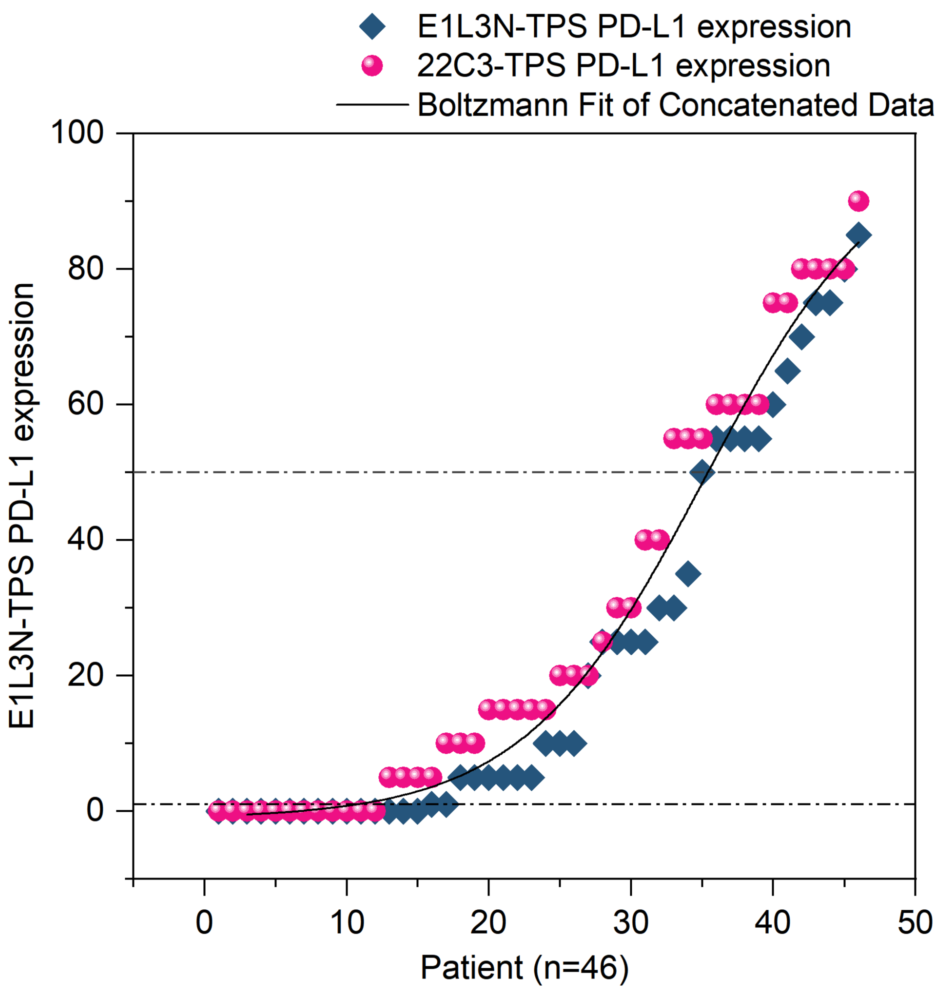

Supplementary Figure 2. Correlation curve plotting the PD-L1 tumor proportion score (TPS) of each patient for 22C3 assay (pink dots) and E1L3N assay (dark blue diamonds) to visualize the distribution of PD-L1 TPS for 22C3 and E1L3N. The dotted lines indicate the cutoff of 1–49%.

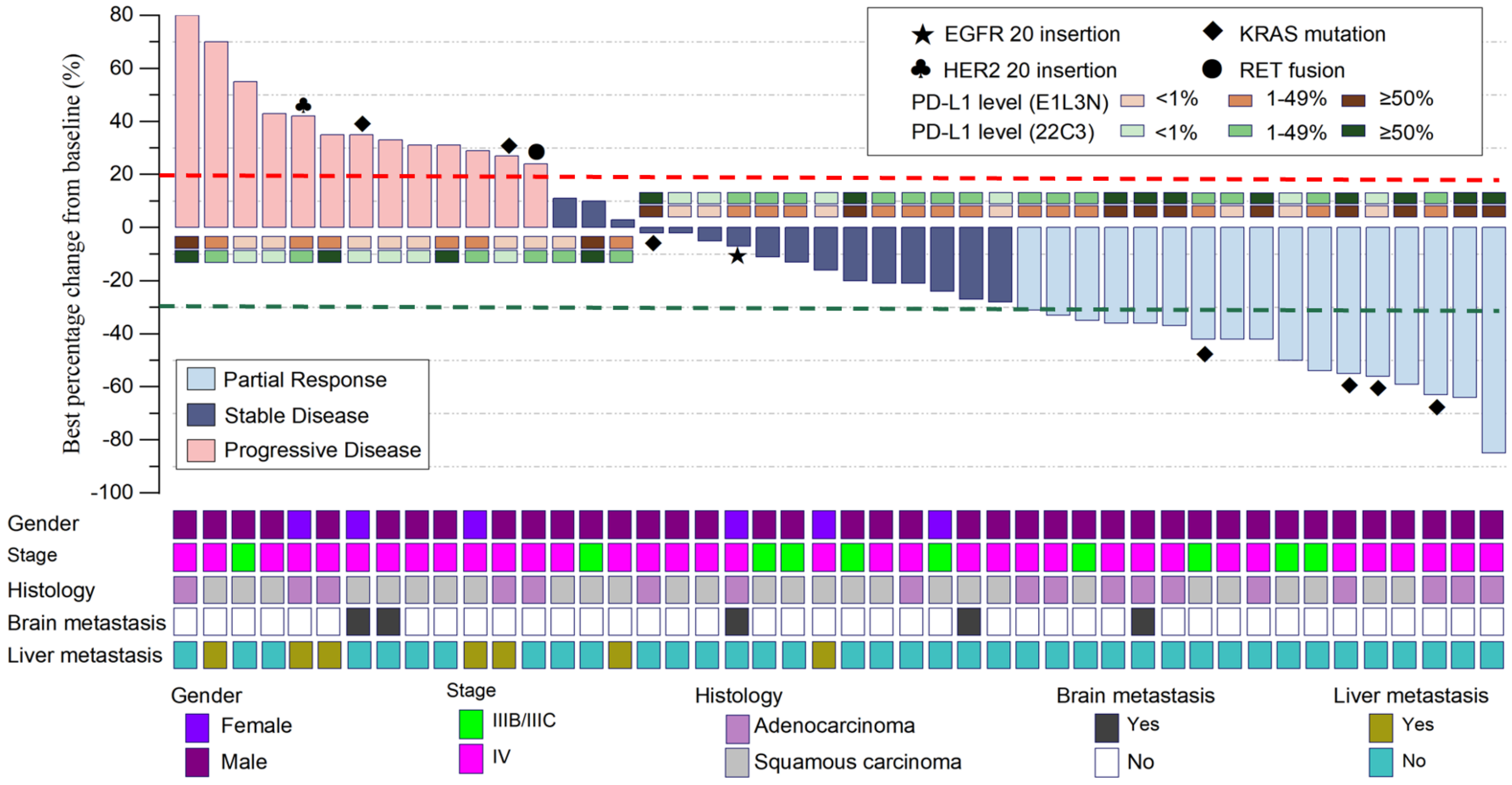

Supplementary Figure 3. Waterfall plot summarizing the best change of tumor size relative to baseline for the whole cohort. Best response, molecular alterations, and PD-L1 expression levels using E1L3N and 22C3 assays were annotated for each patient and represented by colors or symbols. Clinical details of each patient were annotated at the bottom of the waterfall plot and represented by different colors as indicated in the figure legends. Red dotted line indicates the tumor size change of +20% as the cutoff for evaluating progressive disease. Green dotted line indicates the tumor size change of -30% as the cutoff for evaluating partial response.

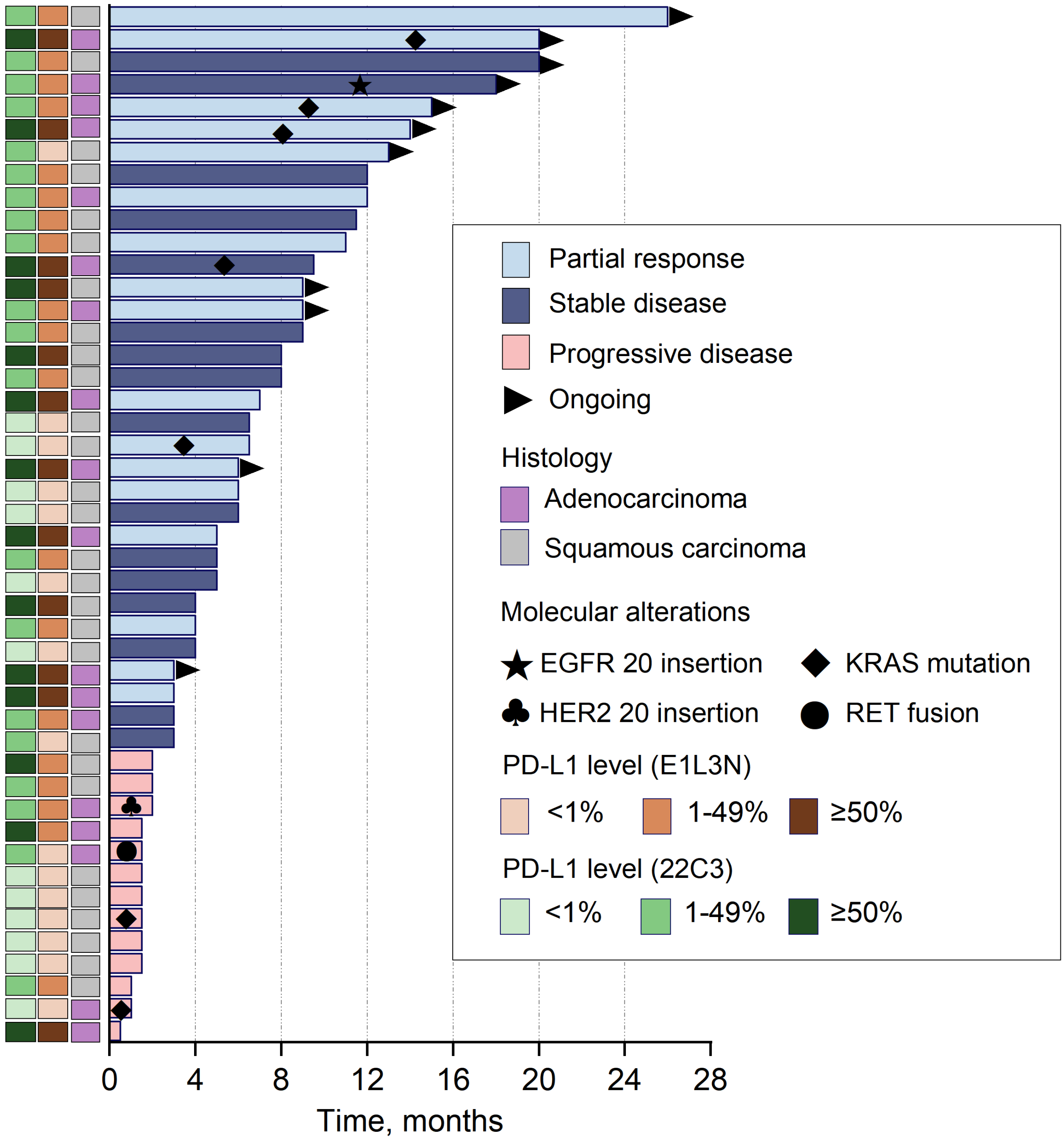

Supplementary Figure 4. Swimmers plot summarizing the progression-free survival of each patient. Best response, histology, molecular alterations, and PD-L1 expression levels using E1L3N assay and 22C3 assays were annotated for each patient and represented by colors or symbols as indicated in the legend.
