# Supplementary material for: Validation of E1L3N antibody for PD-L1 detection and prediction of pembrolizumab response in non-small-cell lung cancer
Source: Commun Med (Lond). 2022 Nov 1;2:137. doi: 10.1038/s43856-022-00206-4 (PMC9626637; doi:10.1038/s43856-022-00206-4)
Supplement: Supplementary file 6 — Description of Additional Supplementary Files [file 43856_2022_206_MOESM6_ESM.pdf]

Description of Additional Supplementary Files

File Name: Supplementary Data 1

Description: Supplementary Data 1 tabulates the PD-L1 TPS for both assays for each of the 46 patients

File Name: Supplementary Data 2

Description: Supplementary Data 2 was the source data for main Figure 3.

File Name: Supplementary Data 3

Description: Supplementary Data 3 was the source data for main Figure 4.

File Name: Supplementary Data 4

Description: Supplementary Data 4 was the source data for main Figure 5
