# Supplementary material for: Validation of E1L3N antibody for PD-L1 detection and prediction of pembrolizumab response in non-small-cell lung cancer
Source: Commun Med (Lond). 2022 Nov 1;2:137. doi: 10.1038/s43856-022-00206-4 (PMC9626637; doi:10.1038/s43856-022-00206-4)
Supplement: Supplementary file 7 — Reporting Summary [file 43856_2022_206_MOESM7_ESM.pdf]

nature research

Corresponding author(s): Yongchang Zhang
Last updated by author(s): Sep 18, 2022

Reporting Summary

Nature Research wishes to improve the reproducibility of the work that we publish. This form provides structure for consistency and transparency in reporting. For further information on Nature Research policies, see our Editorial Policies and the Editorial Policy Checklist.

Statistics

For all statistical analyses, confirm that the following items are present in the figure legend, table legend, main text, or Methods section.

n/a	Confirmed	
☐	☒	The exact sample size (*n*) for each experimental group/condition, given as a discrete number and unit of measurement
☐	☒	A statement on whether measurements were taken from distinct samples or whether the same sample was measured repeatedly
☐	☒	The statistical test(s) used AND whether they are one- or two-sided<br>*Only common tests should be described solely by name; describe more complex techniques in the Methods section.*
☐	☒	A description of all covariates tested
☐	☒	A description of any assumptions or corrections, such as tests of normality and adjustment for multiple comparisons
☐	☒	A full description of the statistical parameters including central tendency (e.g. means) or other basic estimates (e.g. regression coefficient) AND variation (e.g. standard deviation) or associated estimates of uncertainty (e.g. confidence intervals)
☐	☒	For null hypothesis testing, the test statistic (e.g. *F*, *t*, *r*) with confidence intervals, effect sizes, degrees of freedom and *P* value noted<br>*Give P values as exact values whenever suitable.*
☒	☐	For Bayesian analysis, information on the choice of priors and Markov chain Monte Carlo settings
☐	☒	For hierarchical and complex designs, identification of the appropriate level for tests and full reporting of outcomes
☐	☒	Estimates of effect sizes (e.g. Cohen's *d*, Pearson's *r*), indicating how they were calculated

Our web collection on statistics for biologists contains articles on many of the points above.

Software and code

Policy information about availability of computer code

Data collection	No software and code was used.
Data analysis	No software and code was used.

For manuscripts utilizing custom algorithms or software that are central to the research but not yet described in published literature, software must be made available to editors and reviewers. We strongly encourage code deposition in a community repository (e.g. GitHub). See the Nature Research guidelines for submitting code & software for further information.

Data

Policy information about availability of data

All manuscripts must include a data availability statement. This statement should provide the following information, where applicable:
- Accession codes, unique identifiers, or web links for publicly available datasets
- A list of figures that have associated raw data
- A description of any restrictions on data availability

Source data is available as Supplementary Data 1 and available as Supplementary Data 2, 3, and 4 for Figures 3–5. Other data (such as imaging data) are available from the corresponding author upon reasonable request.

Field-specific reporting

Please select the one below that is the best fit for your research. If you are not sure, read the appropriate sections before making your selection.

☒ Life sciences ☐ Behavioural & social sciences ☐ Ecological, evolutionary & environmental sciences

For a reference copy of the document with all sections, see nature.com/documents/nr-reporting-summary-flat.pdf

Life sciences study design

All studies must disclose on these points even when the disclosure is negative.

Sample size	A total of 1,074 patients who were diagnosed with NSCLC between January 2014 and November 2021 at Hunan Cancer Hospital, China were screened for eligibility. Eligible patients were of ≥18 years of age with histologically or cytologically confirmed locally advanced or metastatic NSCLC without actionable mutations. Patients were excluded if tissue samples had inadequate tumor cells (<100 total number of tumor cells) on pathological evaluation. Patients who were being treated with pembrolizumab and neoadjuvant therapy or adjuvant therapy were excluded. Finally, 46 patients were enrolled in this study.
Data exclusions	Patients without tissue samples or who were being treated with pembrolizumab and neoadjuvant therapy or adjuvant therapy were excluded.
Replication	The replication was not relevant.
Randomization	Randomization is not relevant to our study.
Blinding	The blinding was not relevant.

Reporting for specific materials, systems and methods

We require information from authors about some types of materials, experimental systems and methods used in many studies. Here, indicate whether each material, system or method listed is relevant to your study. If you are not sure if a list item applies to your research, read the appropriate section before selecting a response.

Materials & experimental systems

n/a	Involved in the study	
☒	☐	Antibodies
☒	☐	Eukaryotic cell lines
☒	☐	Palaeontology and archaeology
☒	☐	Animals and other organisms
☒	☐	Human research participants
☐	☒	Clinical data
☒	☐	Dual use research of concern

Methods

n/a	Involved in the study	
☒	☐	ChIP-seq
☒	☐	Flow cytometry
☒	☐	MRI-based neuroimaging

Clinical data

Policy information about clinical studies

All manuscripts should comply with the ICMJE guidelines for publication of clinical research and a completed CONSORT checklist must be included with all submissions.

Clinical trial registration	Clinical trials registration: NCT05129462
Study protocol	https://clinicaltrials.gov/ct2/show/NCT05129462?term=NCT05129462&draw=2&rank=1
Data collection	Our study analyzed 46 patients diagnosed with unresectable EGFR/ALK/ROS1-negative NSCLC from 2018 to 2021. PD-L1 immunohistochemistry (IHC)-based assays were performed in baseline tissue biopsy samples using PDL1-E1L3N and PDL1-22C3 antibodies. All patients received first-line pembrolizumab monotherapy. We assessed the concordance between the IHC assays and the treatment outcomes of patients.
Outcomes	We assessed the concordance between the IHC assays and the treatment outcomes of patients.
